# Frequent callers to UK ambulance services in the COVID-19 pandemic: managing mental health, social isolation and loneliness

**DOI:** 10.29045/14784726.2021.09.6.2.66

**Published:** 2021-09-01

**Authors:** Jason Scott, Helen Burtrand, Tim Churchill, Robert Cole, Tracy Collins, Nathan Daxner, Gayle Fidler, Jonathan Hammond-Williams, Benjamin Marlow, Angela McNally, John O’Keefe, Robin Petterson, Deborah Powell, Stephanie Scott, Jayne Scaife, Joanna Smylie, Annette Strickland

**Affiliations:** Northumbria University; East of England Ambulance Service NHS Trust; South Central Ambulance Service NHS Foundation Trust; West Midlands Ambulance Service NHS Foundation Trust; Northumbria University; South East Coast Ambulance Service NHS Foundation Trust; North East Ambulance Service NHS Foundation Trust; South Western Ambulance Service NHS Foundation Trust; South East Coast Ambulance Service NHS Foundation Trust; North West Ambulance Service NHS Trust; London Ambulance Service NHS Trust; Welsh Ambulance Services NHS Trust; East Midlands Ambulance Service NHS Trust; Newcastle University; Scottish Ambulance Service; Northern Ireland Ambulance Service; Yorkshire Ambulance Service NHS Trust

**Keywords:** COVID-19, mental health, social isolation

## Abstract

**Objectives:**

Patients who frequently call ambulance services are a vulnerable yet heterogeneous population with unmet multiple and complex physical health, mental health and/or social care needs. In this article, we report the challenges that the COVID-19 pandemic has introduced for ambulance services across the UK when managing frequent callers, and reflect on how existing systems and practices are adapting to support changing patient needs.

**Methods:**

Data reported in this article comprise reflections from the frequent caller leads in each ambulance service in the UK. All data were provided between 23 April 2020 and 1 May 2020, shortly after the peak of the outbreak in the UK. A single anonymised case study is also reported to illustrate how the pandemic is affecting people’s circumstances and contributing to frequent caller behaviour.

**Results:**

Ambulance services are observing changes to the frequent caller population, with many new frequent callers due to health anxiety caused or exacerbated by the pandemic. Management of frequent callers is also changing, with multidisciplinary and multi-agency working becoming more challenging due to decreased access to external services, whether in social care or the community and voluntary sector, and the redeployment of ambulance service staff. There is also decreased face-to-face contact with frequent callers, meaning that opportunities to deliver person-centred care are reduced. However, the introduction or increased use of tele/video conferencing with other organisations has mitigated some of these challenges, and in some cases has improved engagement among external organisations.

**Conclusions:**

Health anxieties, lack of access to other health, social and community and voluntary sector services and exacerbations of social isolation and/or loneliness have reportedly contributed to changing behaviour among frequent callers. The COVID-19 pandemic has also affected how ambulance services have been able to manage frequent callers. Ambulance services should continue to engage with external organisations to aid the delivery of person-centred care, particularly organisations with experience in multiple complex needs such as mental health, social isolation and/or loneliness. Future research should examine the consequences of the pandemic for frequent users of ambulance services, and how these impact on the wider health and care community.

## Introduction

People who frequently call ambulance services have historically done so because they have unmet multiple and complex physical health, mental health and/or social care needs (Scott et al., 2014a, 2014b). Social isolation and loneliness have been identified as significant contributors to this behaviour (Agarwal et al., 2019) as well as being contributors to non-frequent use of ambulance services (Booker et al., 2019). The emerging COVID-19 pandemic, caused by the severe acute respiratory syndrome coronavirus (SARS-CoV-2), has resulted in unprecedented social change involving locking down large sections of society and social distancing, among a myriad of other policy interventions (Flynn et al., 2020). These measures have the potential to exacerbate many of the problems that contribute to frequent use of ambulance services, such as loneliness, isolation (Armitage & Nellums, 2020) and poor mental health (Yao et al., 2020). In this article, we reflect on perceived changes to frequent caller behaviour, report the challenges that the pandemic has introduced for ambulance services across the UK when managing frequent callers and reflect on how existing systems and practices are adapting to support changing patient needs during the pandemic response.

## Methods

### Description of setting

Ambulance services in the UK deliver care across both rural and urban areas, with the exception of the London Ambulance Service NHS Trust, which does not cover rural locations. As a result of the COVID-19 pandemic, emergency calls to ambulance services initially increased year on year in March but then decreased in April, May and June (Flynn et al., 2020).

The number of people who frequently call UK ambulance services – defined as an individual who calls five or more times in a month or 12 or more times in 3 months – averages around 600–900 per month per ambulance service, though reporting of call volume varies by service despite having a national definition of frequent use (Snooks et al., 2019). Of these, approximately 100 are newly identified each month and the others are pre-existing frequent callers. Each ambulance service generally has some form of proactive management of frequent callers via a dedicated frequent caller team, which follows a best practice guide (available upon request from the corresponding author) for managing frequent callers. The guide includes a four-stage management process following identification of a frequent caller: (1) assessment of patient needs, (2) intervention, (3) evaluation of effectiveness of intervention and (4) escalation. The specific intervention is based on the needs of the individual frequent caller, and can include a letter to the patient and their GP; referral into alternative specialist services, such as other health and social care services or the community and voluntary sector (CVS); and formal clinical case management (Edwards et al., 2015). Frequent callers are then discharged from the care of the frequent caller team once they have no longer met the threshold for a period of 6 months.

### Data sources and analysis

Data reported in this article comprise reflections from the frequent caller leads in each ambulance service in the UK, except for the Isle of Man Ambulance Service, the Isle of Wight Ambulance Service, the States of Jersey Ambulance Service and the Guernsey Ambulance and Rescue Service. These services were not included as they are not active members of the Frequent Caller National Network, which is a formal ambulance service group that aims to improve the quality of care for vulnerable patients and those with complex needs who frequently call ambulance services. The frequent caller lead for each service, also co-authors, completed the relevant sections of Table 1. This included perceived changes to frequent caller behaviour as a result of the pandemic, changes made to practice and perceived impact of the changes on service provision. All data were provided between 23 April 2020 and 1 May 2020, shortly after the peak of the outbreak in the UK. A single anonymised case study is also reported to contextualise how the pandemic is affecting frequent caller behaviour. The case study is not intended to reflect a ‘typical’ frequent caller, but rather to illustrate how the COVID-19 pandemic has changed people’s circumstances and led to frequent calling. The results are reported narratively.

**Table 1.** COVID-19 pandemic impact on frequent caller behaviour and ambulance service practice.

Ambulance service	Reflections on frequent caller behaviour	Challenges in management and adaptations to practice
**England**		
East of England Ambulance Service NHS Trust	A large number of newly calling patients have been triggering the frequent caller criteria since the start of the lockdown.	No changes have been made as of yet to the management of frequent callers policy, but the majority of the frequent caller team have been redeployed to front-line roles.
East Midlands Ambulance Service NHS Trust	There has been little change with entrenched frequent callers. Newly identified frequent callers seem to have health anxiety exacerbated by social isolation and lack of purpose.	Some professionals from other services that this ambulance service work in partnership with have been redeployed to front-line roles. This has reduced the ambulance service’s ability to put in place collaborative care plans.
London Ambulance Service NHS Trust	Frequent caller cohort divided into three groups: (1) Current frequent callers whose behaviour did not change. This was a lower than normal number. (2) Current frequent callers who reduced or stopped calling. (3) New callers who were experiencing health anxiety. Caller numbers overall went down, but calls went up. The number of deaths among frequent callers also rose.	Referrals and signposting to other services are continuing, but there is a delay in responses from these other services due to staff self-isolating and social distancing. Meetings with external services have moved to tele/video conferencing or been cancelled. In some cases, for instance with General Practitioners, there is improved engagement. Face-to-face visits with frequent callers have stopped, possibly impacting on effectiveness of interventions. Some multidisciplinary team tele/video conferences have occurred including patient participation. Letters to current frequent callers whose behaviour has not changed will continue to be sent. Patients are reviewed beforehand for appropriateness. Health anxiety callers are not actively managed but reviewed after six weeks to determine whether behaviour was acute due to the pandemic.
North East Ambulance Service NHS Foundation Trust	Decline in known frequent callers, however there is an apparent rise in new frequent callers in response to the pandemic.	New frequent callers are registered and monitored. The ambulance service is currently looking to provide clinician call-backs with mental health support once time allows, to prevent frequent calling behaviour from becoming embedded.
North West Ambulance Service NHS Trust	A small number of patients previously identified as frequent callers with high call volumes have reduced their call volume, and a small number have ceased calling. A new cohort of patients has hit the frequent caller thresholds since the start of the pandemic. Some of the increase appears to be due to the pandemic where there has been a change in daily routine, causing distress to patients who have not previously been known to frequently access 999.	External meetings are taking place by means of tele/video conference where previously these have taken place face to face. Having this IT platform supports the on-going multi-agency work to support patient well-being. Visiting patients in their home environment has ceased since lockdown. The frequent caller team are carrying out telephone welfare calls to frequent callers who are already known to the team (majority of whom are known to live alone) to support, advise and signpost where required to council services support hubs. New frequent callers identified as 70 years or over and/or vulnerable are being contacted (where capacity allows) to signpost accordingly. Places of residence and patients detected by the frequent caller team as new frequent callers will be reviewed to identify if any are: Child frequent callers (below 18 years of age); Adult frequent callers who are 70 years of age and above. Safeguarding concerns will be processed following normal safeguarding procedures. This will include children identified in the household of a frequent caller where safeguarding concerns have also been identified for the child. The frequent caller team is working more closely with Emergency Operations Control Centre colleagues to support earlier identification of patients at risk of becoming frequent callers. This work is in its infancy and progressing within the service to support COVID-19 crisis management and patient care.
South Central Ambulance Service NHS Foundation Trust	The service has seen an escalation in call volume since 23 March from some known frequent callers, particularly those whose presentations are learning disability, Autistic Spectrum Disorder and Attention Deficit Hyperactivity Disorder. Talking with some of these patients, it appears to be driven by anxiety and disruption to their normal routines (particularly the removal of planned support and face-to-face visits from other services). For some other frequent callers, there has been a reduction in activity.	Demand Practitioners are having increased interaction with patients in the given cohort, with patient care plans being revised to take account of the reduction in community support. Demand practitioners support the management of frequent callers by identifying the root cause of calling behaviour, support patients in navigating health and care systems, organise and lead multidisciplinary working, develop management plans and monitor and review frequent callers.
South East Coast Ambulance Service NHS Foundation Trust	Pre-existing callers have increased their call volume, with a few noticeable examples of increased anxiety. This may be linked to a disruption to the patients’ usual routine where lockdown has meant family/friends cannot visit as usual and care agencies have been required to adapt their working practices. In May, the cohort of patients increased by 100 to 240 newly identified frequent callers across the region, 48 of these primarily calling for COVID concerns (either suspected/confirmed) or for general anxiety. On top of this there has been a marked increase in anxiety/social-related calls.	The management of frequent callers has not changed, with patients still receiving written correspondence where required. External meetings have been moved to tele/video conferencing where facilities allow. We have noticed an increase in attendance of these as invitees can dial in from home. Home visits to conduct a face-to-face consultation have been cancelled, therefore the opportunity to see the patient in their home environment and review their medical/holistic needs is missed. This may have an impact on referrals to external services such as social services, occupational therapy and social prescribing. The frequent caller team are actively managing those patients who are new with health anxieties or those presenting with COVID-19 symptoms. During the COVID-19 pandemic the frequent caller team have been able to complete their work remotely from home without being redeployed.
South Western Ambulance Service NHS Foundation Trust	Majority of patients known to the frequent caller team have stopped calling. These patients have been replaced with >1100 frequent callers not previously known. A review of patients indicates this is due to a breakdown in their normal coping strategies and support network.	Productivity has reduced; all the team are now working from home. This means the team commonly have less hardware compared to when working within the Clinical Hub. Also having so many new patients to review, with no previous knowledge of them, means it is more time consuming to review and collaborate about each individual. The Trust has continued to invest in the development of a new electronic management system to assist data collection and analysis of the number of calls, presenting complaints and outcomes of the activity generated by patients that meet the national definition of a frequent caller. Both internal and external meetings have changed to video conference calls. Some commissioner-level meetings have stopped due to the pandemic, meaning other priorities have taken precedence. The Frequent Caller Lead is involved in daily briefings about COVID-19 and highlights risks and opportunities with good support from the Trust’s Strategic Team. The introduction of the Pandemic protocol within the Medical Priority Dispatch System (MPDS) has also seen the development of a local standard operating procedure to work alongside the pandemic protocol within the MPDS to ensure that triage of frequent callers is still appropriate in line with recommendations following assessment of the frequent caller’s clinical need and risk, while still enabling a safe and efficient method to recognise COVID-19 symptoms and assess and respond appropriately to all.
West Midlands Ambulance Service NHS Foundation Trust	There has been a mixed response, with some frequent callers having stopped calling altogether, but most have increased their call volume, of which a majority of calls are for welfare and assistance. The effects of lockdown are being seen with regard to individuals’ mental ill health, and in certain cases the inability to leave the house has significantly increased anxiety and stress responses.	The ability to undertake multidisciplinary team meetings, even remotely, has been reduced. This is often due to the redeployment of key staff, which has an adverse effect on frequent callers as they are not having their individual (and altered) needs met, which results in a worsening of the situation for all. Patients’ physical and mental health may deteriorate, and escalating behaviours can lead to increased demand.
Yorkshire Ambulance Service NHS Trust	The service has identified around double the number of frequent callers who are calling above baseline since March. A review has observed a mix of new patients who are new to service or existing patients that have had a breakdown of support systems in the community.	Initially, half of the frequent caller team were redeployed to telephone triage while the remaining team was asked to a) identify on a daily basis callers that had contacted three or more times in the last 24 hrs b) conduct welfare calls on existing frequent callers who were high risk, shielding or known to be over 70, and signpost them to local COVID-19 mutual aid groups.
**Northern Ireland**		
Northern Ireland Ambulance Service	The daily call volume from known frequent callers has not changed. However, the number of new frequent callers has increased by nearly a quarter of the current total since the beginning of March.	Letters were sent to the top active callers in mid-March reminding them of the risks due to inappropriate ambulance use during the pandemic. The top 20 frequent callers in call volume are reviewed fortnightly and appropriate information markers are placed on addresses following risk assessment.
**Scotland**		
Scottish Ambulance Service	The service is not yet regularly monitoring a regular frequent caller data set, however during case management of some individuals it has been noted that contact to the service has increased during the course of the pandemic. This has been found to be due either to a breakdown in current supportive care pathways or to new and emerging mental and emotional health problems caused by the social isolation.	Monitoring and management of frequent callers on a regular basis have not yet begun in this service, and so there are no evidential changes to report. The service is in the process of developing management strategies based on discussions with other UK ambulance services. Due to the pandemic this work has been placed on hold, meaning there is a disconnection between some local health care providers and the ambulance service. As the pandemic promotes improved unity in ways of delivering and managing care, these issues will slowly be resolved on a case-by-case basis, which will support the need for a true integrated model of care delivery.
**Wales**		
Wales Ambulance Service	A number of patients previously identified as frequent callers with high call volumes have reduced their call volume. Some have even stopped calling. There has been an increase in frequent callers in some Health Boards, while in others the demand has appeared to remain the same. A new, small cohort of patients now seems to be appearing as frequent callers. Some elderly patients have been contacting and speaking with family members; their concerns appear to be related to self-isolation and that they are no longer attending day hospitals and receiving the support and contact this has provided them. There are also a number of patients now appearing who are known to mental health services but are ringing due to social health issues such as drinking alcohol more heavily.	The entire frequent caller team have been pulled from managing frequent callers, however in their new roles they have managed to signpost patients to General Practitioners who are happy to provide advice or support during the current pandemic.

Notes: Data within the table were generated by respective co-authors from each service. This includes all ambulance services in the UK except for the Isle of Man Ambulance Service, the Isle of Wight Ambulance Service, the States of Jersey Ambulance Service and the Guernsey Ambulance and Rescue Service.

## Results

### COVID-19 challenges and adaptations to practice

Ambulance service frequent caller leads have identified a perceived increase in the number of patients classified as a frequent caller (see Table 1 for a summary of service-by-service descriptive changes to frequent caller behaviour). While frequency of calling as a categorisation marker results in a heterogeneous group, there is a pattern emerging where existing (pre-pandemic) frequent callers either are continuing their behaviour, or in some cases have reduced or ceased calling, though quantitative data on call patterns is not yet available. Services also report identifying an increased number of new frequent callers, the majority of whom appear to be doing so due to health anxiety caused or exacerbated by the pandemic. These patients are reportedly experiencing changes to daily routines and a lack of engagement in meaningful occupations, and have been struggling to access other non-emergency services (see anonymised case study below). To respond to this challenge, some services have begun monitoring new frequent callers to determine whether their behaviour will become persistent once lockdown requirements ease, with the intention of starting mental health and welfare calls in an attempt to reduce escalation.

As well as patients struggling to access existing services in social care and the CVS, ambulance services managing patients are experiencing similar challenges, particularly in their ability to deliver clinical case management. For instance, some frequent caller teams have reported experiencing delays in response from external services, especially those with a high number of volunteers who may themselves be at risk due to comorbidities and/or age group. Several frequent caller teams have also had their own capacity reduced where staff are re-deployed to the front line to cope with the increase in demand. Reduced face-to-face meetings with frequent callers as a result of social distancing requirements has also resulted in reports of reduced person-centred care. There has also been an increase in remote working and use of teleconferencing to meet with other agencies, which for one service has positively resulted in increased attendance from General Practitioners (GPs).

## Case study illustrating how changes to a person’s circumstances as a result of the COVID-19 pandemic can result in frequent calling

An adult patient with Autistic Spectrum Disorder was recently discharged from hospital. Appropriate discharge planning, including provision of suitable activities, had been made prior to discharge and the patient initially settled in well to the new routine and accommodation. Due to COVID-19 restrictions, the patient’s planned routine and structure were unable to continue, which resulted in ‘meltdowns’ and calling on emergency services for help. When the patient experienced a ‘meltdown’, he became non-verbal and could only communicate through vocal stereotypy, which resulted in confusion for the emergency services call takers. Rapid action was taken by services involved to arrange a multidisciplinary meeting to discuss further support.

The patient was receptive to receiving support from relevant services and prior to the current COVID-19 situation did not call 999. Having had the relevant discussions with all services involved, support services have now increased telephone contact to chat, reassure and support the patient. A smartphone and tablet are being arranged so the patient can access the Brain in Hand app which links up to the National Autistic Society when the patient is in crisis. Information is now readily available to emergency services regarding the patient’s usual presentation and recent concerns. The information also provides contact details for professional support networks for clinicians to call as a pathway of support and referral. This aims to provide the appropriate support delivered by the relevant provider in the right place to support patient need, which in turn prevents inappropriate emergency services deployment.

## Discussion

This short report provides reflections on how the COVID-19 pandemic is influencing frequent caller behaviour, the challenges this introduces for UK ambulance services and how these are being addressed. Health anxiety and lack of access to other services are reported as key drivers of frequent calling behaviour, along with social isolation and loneliness. Social distancing and lockdown altered daily routines, as demonstrated in the presented case study, which can exacerbate loneliness and social isolation as a result of a lack of engagement in meaningful occupations (Collins et al., 2020). There has been a reported impact on how services manage frequent callers, particularly clinical case management that includes multidisciplinary team meetings and multi-agency collaboration. Given that this is a typical approach to managing frequent callers (Edwards et al., 2015), it likely means that the quality of care delivered to frequent callers has been suboptimal, at a time where due to the complex nature of their care needs they arguably needed support the most.

The results reported in this short report make it clear that there is a need to further study the impact of the COVID-19 pandemic, including societal changes such as lockdown and social distancing, on frequent callers to ambulance services. To our knowledge there is no ongoing research into the impact of the pandemic on frequent callers. Therefore, future research should quantitatively examine how the epidemiology of frequent use has changed, including whether these changes become entrenched or whether they return to pre-pandemic trends, to inform future practice. One potential avenue would be to explore whether telemedicine solutions could be utilised to engage with frequent callers, including whether such an intervention would digitally exclude those with little or no internet connection. There is also a need for qualitative research to examine the perspective of frequent callers to understand such changes in more depth, with a particular emphasis on how wider societal changes have contributed or not. This can go some way to helping us understand the phenomenon of frequent use, which is still poorly understood (Scott et al., 2014b), and adapt interventions post-pandemic.

The strategy, policy and practice adaptations identified in this short report provide potential solutions for other emergency services within both the UK and internationally which are dealing with increased calls relating to mental health, social isolation and loneliness, and are having to adapt to social distancing restrictions. The use of technology to facilitate multidisciplinary and multi-agency working is important. Telehealth and video consultations, which have seen significant attention in response to the pandemic (Smith et al., 2020), could be explored as a temporary or permanent solution to reduced face-to-face contact with frequent callers. Nevertheless, it is unlikely that the pandemic will impact all people in a uniform way, with social distancing measures likely to result in deepening inequalities, and vulnerable populations and people on low incomes most prone to adverse effects (Douglas et al., 2020). Ambulance services should continue to explore and engage with CVS organisations around how they can engage with patients, particularly those CVS organisations with experience in mental health, social isolation and/or loneliness. This will be particularly important should the pandemic continue for the medium to long term with regionalised or national outbreaks.

## Conclusions

Health anxieties, lack of access to other health, social and CVS services and exacerbations of social isolation and loneliness have reportedly contributed to changing behaviour among frequent callers. Ambulance services should continue to engage with external organisations to aid the delivery of person-centred care, particularly organisations with experience in multiple complex needs such as mental health, social isolation and/or loneliness. Future research should examine the consequences of the pandemic for frequent users of ambulance services, and how these impact on the wider health and care community.

## Acknowledgements

We would like thank Juliette Smyth and Chris Williams (London Ambulance Service NHS Foundation Trust) for reviewing and commenting on a draft of the article.

## Author contributions

JSco conceived the article idea. HB, TC, RC, ND, GF, JHW, BM, AM, JO, RP, DP, JSca, JSm and AS contributed practice perspectives. All authors contributed to drafting the manuscript and revising it critically for important intellectual content. JSco acts as the guarantor for this article.

## Conflict of interest

None declared.

## Funding

None.
